# Tolerance of preterm formula versus pasteurized donor human milk in very preterm infants: a randomized non-inferiority trial

**DOI:** 10.1186/s13052-018-0532-7

**Published:** 2018-08-16

**Authors:** Simonetta Costa, Luca Maggio, Giovanni Alighieri, Giovanni Barone, Francesco Cota, Giovanni Vento

**Affiliations:** Department of Woman and Child Health, Obstetric and Neonatology Area, Fondazione Policlinico Universitario A. Gemelli IRCCS, Largo A. Gemelli 8, 00168 Rome, Italy

**Keywords:** Feeding tolerance, Pasteurized donor human milk, Preterm formula, Very preterm infant

## Abstract

**Background:**

Human milk (HM) is the best feeding for premature infants. When own mother’s milk (OMM) is insufficient or unavailable, pasteurized donor human milk (PDHM) and preterm formula (PF) are the alternative nutritional sources, but the benefits of donor milk over formula are not defined. This study aimed to assess whether, in the absence of OMM, the PF could guarantee a feeding tolerance not inferior to that seen with the use of PDHM during the first two weeks of life of very preterm infants.

**Methods:**

Infants with gestational age (GA) of ≤32 weeks who started enteral feeding within the first 7 days of life were randomized to receive PDHM or PF as a supplement to the OMM insufficient or unavailable. The primary outcome was the day of life when full enteral feeding (FEF) of 150 mL/Kg/d was achieved.

**Results:**

Seventy infants were randomized, 35 in the PF group (GA 30.2 ± 1.7 weeks; BW 1342 ± 275 g), 35 in the PDHM group (GA 30 ± 1.9 weeks; BW 1365 ± 332 g). The time to achieve FEF was the same for infants fed with PF and for infants fed with PDHM (12.3 ± 7.0 days vs 12.8 ± 6.5).

**Conclusions:**

This trial shows that PF could be a valid alternative for the early feeding of very preterm infants when OMM is insufficient or unavailable.

**Trial registration:**

UMIN000013922. Date of formal registration: December 31, 2014.

## Background

Human milk (HM) is the best feeding for infants, especially for the premature high-risk infants [1, 2].

After preterm delivery, the own mother’s milk (OMM) is not always available because many mothers produce insufficient milk to meet their infant’s needs. It has been found that only 27% of mothers were able to sustain their lactation to meet the needs of their premature infants throughout the hospitalization [3]. Results from an Italian study showed that 55.8% of infants with BW of < 1500 g discharged from intensive care unit are fed with exclusive, predominant or complementary HM, but only 3.1% of these infants are discharged at home with OMM [4]. Similarly, a survey carried out on 97 United States neonatal intensive care units revealed that 42% of very low birthweight infants receive human milk mixed with fortifier or formula and only 6% are discharged on exclusive human milk [5].

When OMM is insufficient or unavailable, pasteurized donor human milk (PDHM) and preterm formula (PF) are the alternative sources of enteral feeding for premature infants. PDHM is stated as the most appropriate alternative to promote feeding tolerance [6], although it seems that PDHM does not have the same benefits of OMM. This is because the process of pasteurization reduces the content and function of some host defence proteins and cellular elements [3, 7]. Premature infants fed with PDHM appear to have a lower risk of necrotizing enterocolitis (NEC) but slower rates of growth in comparison with those fed with PF [8–12]. A retrospective study by Sisk et al. suggested a strong association between exposure to OMM and PDHM and a lower rate of NEC, without significant differences in growth compared with HM or PF [13], and a randomized controlled study showed no significant effect of PDHM compared to PF for preventing infections, NEC, and mortality [14]. Moreover, PDHM is not always available because HM donation is voluntary and not all hospitals have a HM bank [15, 16].

We hypothesized that, during the first two weeks of life, premature infants fed with PF, as alternative to OMM insufficient or unavailable, would have a feeding tolerance not inferior to that seen in infants fed with PDHM.

## Methods

In this mono-centric, randomized, non-inferiority trial, infants were eligible if they had GA of ≤32 weeks, started enteral feeding within the first 7 days of life, and if OMM was unavailable or insufficient to satisfy the planned enteral intakes. Infants were not enrolled if they had congenital malformations, connatal infections, or abnormal antenatal Doppler flow velocimetry [17]. Infants with antenatal abnormal velocimetry were excluded because they are at higher risk of NEC; for these infants it is recommended to start feeding with HM [18].

The institutional review boards approved the study, and written informed consent was obtained from the parents of all subjects before enrollment.

When enteral nutrition was initiated, enrolled infants were randomized to receive PDHM or PF as a supplement to the OMM insufficient or unavailable. Before randomization, infants did not receive any feeding.

Recruitment of patients started on January 1, 2015 and ended on August 31, 2015 (Clinical Trial Registration: UMIN000013922. Date of formal registration: December 31, 2014).

The PF diet consisted of 3.5 g of protein/100 Kcal formula (Plasmon PreZero, Plasmon, Italy). Our Hospital does not have a HM bank. PDHM for this study came from mothers who prematurely delivered and whose infants were hospitalized in our intensive care unit.

Mothers negative for HIV, HBV, HCV, CMV, Typhoid, Paratyphoid, Brucellosis, Pertussis, active pulmonary Tuberculosis, Lues, and mothers without medication incompatible with breastfeeding were supported since the first day after delivery to milk expression by neonatologist stuff through oral, written and iconographic information. Mothers stored the milk at 4 °C in a dedicated refrigerator, and then milk was pasteurized within 24 h of collection by the Holder method (+ 62.5 °C for 30 min). The milk of mothers who produced a quantity of milk exceeding the needs of their infants, and who decided to donate, was used for infants randomized in the PDHM group. Only the donor HM was pasteurized.

It was assumed that the protein/caloric ratio of the PDHM as well of the OMM was 2.5 g of protein/100 Kcal [19].

The primary outcome of the study was the day of life at which FE F of 150 mL/Kg/d of milk was achieved and well tolerated for at least tree consecutive days. Secondary outcomes were nutritional milestones (days of fasting, days of parenteral nutrition (PN), total of enteral and intravenous fluids intake, total caloric and protein intake in the first 15 days of life), clinical outcomes (late-onset sepsis, NEC, respiratory distress syndrome, patent ductus arteriosus, bronchopulmonary dysplasia, length of hospital stay and mortality) anthropometric outcomes (maximum weight loss, time to regain birthweight; weight, length and head circumference at 14 days of life and at 36 weeks of postmenstrual age (PMA) or at discharge).

Given that our very premature infants fed with PDHM in the last 2 years reached FEF on average at 13 ± 6 day of life, we assumed that a difference > 3 days in the achievement of FEF in the group of infants fed with PF would be clinically relevant to determine the inferiority of PF compared with PDHM. We calculated that 70 patients, 35 for each group, would be required to have an 80% chance of ruling out a difference of 3 days with 95% confidence (one-sided analysis).

Randomization was stratified by GA (< 28/≥28 weeks). For allocation of the participants, a computer-generated list of random numbers was used. The allocation sequence was concealed from the researchers enrolling and from those assessing the infants. A 2-tailed value of *p* < 0.05 was considered significant. Statistical analysis was performed using the “Stata Statistical Software: Release 1” (StataCorp LP, College Station, Tx).

The PN was started soon after birth in all infants with a BW of< 1250 g, and in infants with BW of < 1500 g requiring invasive ventilation.

The PN was stopped when enteral intake was > 125 mL/Kg/d. Minimal enteral feeding was initiated within the first 48 h of life and was continued at 20 mL/Kg/d for up to 5 days. Subsequently, enteral nutrition volume was increased by 20 mL/Kg/d.

The criteria for reduction of enteral feeding were a gastric residual volume more than 2 mL or more than 1/3 of the previous meal volume for 3 consecutive meals, or more than 3 consecutive vomiting. The reduction of the enteral feeding consisted in the administration of a volume of milk equal to that previously tolerated. The criteria for complete withdrawal of enteral feeding were a gastric residual volume bloodstained after a single meal, or bile-stained in the presence of pathologic abdominal signs or poor clinical conditions. Signs of pathological abdomen were: distension with an increase in abdominal circumference greater than 2 cm in 3 h, discoloration or tenderness, visible bowel loops for at least 6 consecutive hours, bloody stool. Poor clinical conditions were considered when infant appeared not well in the presence of metabolic acidosis with pH < 7.20 for more than 2 h or hypoxemia with paO2 < 50 mm/Hg for more than 2 h or hypotension [20].

## Results

**Fig. 1:**
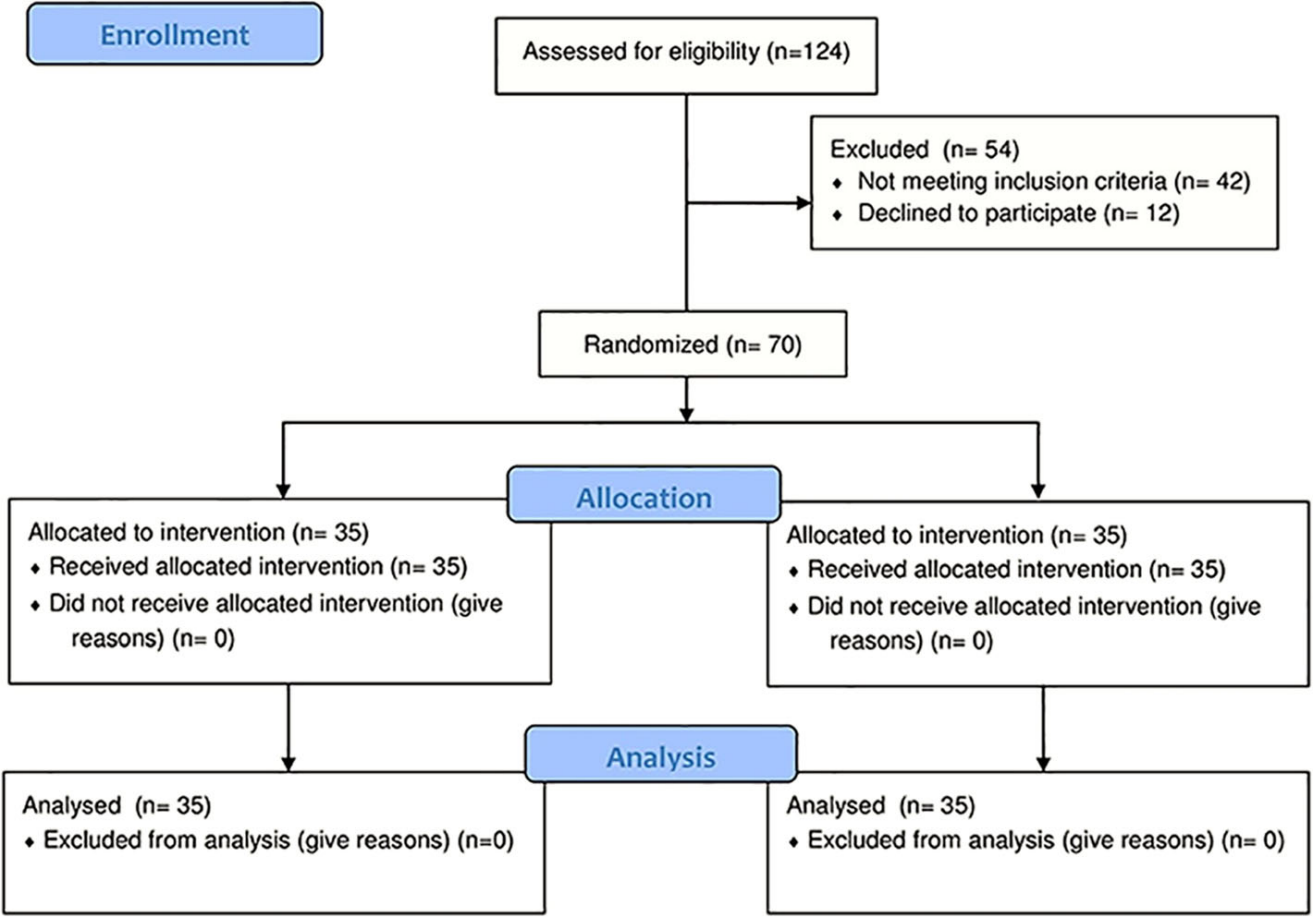
Flow chart of the study

During the study period 124 eligible infants were admitted in our neonatal intensive care unit. We excluded 18 infants with abnormal or unknown foetal Doppler velocimetry, 3 with congenital anomalies, 2 because of connatal infections, and 19 because they did not start enteral feeding within the 7th day of life; 12 infants were excluded because parents declined to participate to the study. The remained 70 preterm infants were enrolled, 35 infants in each group, and complete data were available for all of them (Fig. 1).

Baseline characteristics were well balanced between the 2 groups (Table 1).

**Table 1:** Characteristics of the Patients at Baseline. Data are presented as number (percentage) or mean ± SD

	PF N 35	PDHM N 35	*p*
Gestational age, wk	30.2 ± 1.7	30 ± 1.9	*0.73*
Birthweight, g	1342 ± 275	1365 ± 332	*0.75*
Length at birth, cm	38.7 ± 3.6	38.6 ± 3.0	*0.88*
Head circumference at birth, cm	28.3 ± 2.1	27.5 ± 1.9	*0.29*
Small for gestational age, *n*	4 (11.4)	4 (11.4)	*1*
Antenatal steroids, *n*	29 (83)	30 (86)	*1*
Male gender, *n*	18 (51)	14 (40)	*0.47*

Table 2 reports the primary and secondary nutritional outcomes of the study. Time to achieve the FEF was the same for both groups; infants in the PF group received a significantly higher total caloric and protein intake.

**Table 2:** Primary and secondary nutritional outcomes. Data are presented as mean ± SD

	PF N 35	PDHM N 35	*p*
Time to full enteral feeding, d	12.3 ± 7.0	12.8 ± 6.5	*0.76*
Minimal enteral feeding, d	3.0 ± 2.6	2.6 ± 2.2	*0.52*
Nihil per os, d	2.0 ± 1.7	1.7 ± 1.7	*0.57*
Parenteral nutrition duration, d	9.1 ± 6.8	7.9 ± 6.6	*0.45*
Total fluids intake, mL/Kg/d	142.3 ± 6.3	143.6 ± 7.7	*0.45*
Total intravenous fluids intake, mL/Kg/d	70.6 ± 42.7	64.3 ± 41	*0.52*
Total enteral fluids intake, mL/Kg/d	68.2 ± 41.1	76.5 ± 37.6	*0.29*
Total caloric intake, Kcal/Kg/d	89.4 ± 7.7	79.3 ± 9.6	*< 0.001*
Total protein intake, g/Kg/d	3.2 ± 0.4	2.4 ± 0.9	*< 0.001*
Own mother’s milk, mL/Kg/d	19.1 ± 24.5	23.7 ± 27.6	*0.45*
Own mother’s milk, % of total enteral intake	28	31	*1*
Total PDHM, mL/Kg/d	0.0 ± 0.0	52.8 ± 37.5	*< 0.001*
Total PF, mL/Kg/d	49.1 ± 42.7	0.0 ± 0.0	*< 0.001*

No differences were found in clinical and anthropometric items, but infants in the PF regained birthweight two days earlier than infants in the PDHM (Table 3). One infant in the PDHM group died because of SIDS (sudden infant death syndrome) occurrence the day before the discharge at home.

**Table 3:** Clinical outcomes and anthropometric items of the study infants. Values are presented as mean ± SD or number (percentage)

	PF N 35	PDHM N 35	*p*
Sepsis, *n*	2 (5.7)	5 (14.3)	*0.42*
NEC, *n*	0	0	*1*
RDS, *n*	17 (48.6)	23 (65.7)	*0.22*
PDA, *n*	10 (28.6)	8 (22.8)	*0.78*
BPD, *n*	1 (2.8)	1 (2.8)	*1*
Length of hospital stay, d	37.5 ± 17.9	39.3 ± 18.6	*0.68*
Mortality, *n*	0	1 (2.9)	*1*
Maximum weight loss, %	12.6 ± 4.5	11.9 ± 5.1	*0.55*
Time to regain birthweight, d	12.9 ± 2.6	15.0 ± 5	*0.032*
Weight at 15 days of life, g	1395 ± 268	1354 ± 327	*0.57*
Length at 15 days of life, cm	40.5 ± 2.6	40.4 ± 2.9	*0.91*
Head circumference at 15 days of life, cm	28.4 ± 2.1	28.1 ± 2.1	*0.57*
Weight at 36 weeks of PMA, g	2010 ± 213	2024 ± 438	*0.67*
Length at 36 weeks of PMA, cm	43.2 ± 1.9	43.7 ± 3.1	*0.42*
Head circumference at 36 weeks of PMA, cm	31.0 ± 0.9	31.0 ± 1.6	*0.93*

## Discussion

We found that, if the OMM is insufficient or unavailable, the PF can ensure a feeding tolerance not inferior to that seen with the use of PDHM during the first two weeks of life in very preterm infants.

Feeding intolerance is one of the major problems of prematurity; it results from the necessity to feed infants who are developmentally a second semester fetus ex utero. Symptoms associated with feeding intolerance are increased gastric residual volume leading to vomiting, abdominal distention, failure to stool, and increased apnoea. Feeding intolerance causes disruption of feeding plan and delay in the achievement of FEF [21, 22].

Our findings do not agree with those of Cristofalo et al., whose study had as primary outcome the duration of PN, as a surrogate of feeding tolerance. The Authors found that extremely preterm infants fed exclusive PDHM diet required fewer days of PN than infants fed PF [11]. Two important differences between our study and that of Cristofalo et al. have to be considered: infants in our study were about two weeks more mature than infants in the Cristofalo study, and infants in both PDHM and PF groups received an amount of OMM of 31% and 28%, respectively, of the total enteral intake, while infants in Cristofalo study did not receive at all OMM.

Sullivan et al., who also used the duration of PN as their study’s primary outcome, and as a surrogate of feeding tolerance, found no significant differences for the duration of PN and for the ages of attainment of FEF of 140 mL/Kg/d between the group of infants fed exclusively HM–based diet and the group of infants fed a HM–based diet that also includes bovine milk–based products. As in our study, all infants of Sullivan study received an amount of OMM [10]. Moreover, in both the mentioned studies the diet with PF or with bovine milk-based products was associated with significantly higher rate of surgical NEC.

Our data are consistent with those of Corpeleijn et al., who found that very low birthweight infant, fed with PF during the first 10 days of life if OMM was not sufficient or unavailable, reached an enteral intake of 120 mL/Kg/d at the same median age of those infants fed with PDHM (11 days in the PF group and 10 in the PDHM group) [14]. Furthermore, the Authors found no significant effect of PDHM during the first10 days of life for preventing NEC.

The American Academy of Pediatrics policy statement on the use of HM states that premature infants should receive only milk from their mother and that, if it is not available, PDHM should be used [2]. This statement is based on the evidence that HM is associated with several benefits, including a better feeding tolerance and a lower risk of NEC. A Cochrane review by Quigley and McGuire found that feeding with formula compared with donor breast milk may increase rates of short-term growth in preterm or low birthweight infants but is associated with a doubling of the risk of developing NEC [12]. More recently, Sisk et al. found a strong association between exposure to OMM and PDHM and a lower rate of NEC [13].

However, as already mentioned, not always mothers who prematurely delivered are able to sustain their lactation throughout the hospitalization. There are different reasons why OMM may not be available, such as maternal exposure to medications or medical complications, absence of mother because of distance from hospital, and maternal use of illicit drugs. In addition to this, not all birth centres have the HM bank to meet the needs of PDHM of their patients; in these circumstances the PF could be the nutritional alternative.

We found that PF can ensure a feeding tolerance not inferior to that seen in infants fed PDHM. This was maybe because PF used for the study had some characteristics that make it digestible. The presence of Betapol, a fat blend that includes synthetic triglycerides, so that about 70% of the palmitate was in the Sn-2 position just like the HM, improves fatty acid and calcium absorption, reduces the formation of insoluble calcium soaps and the stool hardness [23]. The formula study had 0.7 g/100 mL of galactooligosaccharides, and 4 mg/100 mL of nucleotides. The galactooligosaccharides are not the same present in HM, but they have been shown to reduce stool viscosity, and to accelerate gastrointestinal transport, facilitating feeding advancement [24]. Formula nucleotide supplementation was shown to have a favourable effect on the gastrointestinal microbiota, which seems to be similar to that of infants fed with HM [25].

This study has some limitations. First, the inclusion criteria, such as the GA of ≤32 weeks and the absence of abnormal antenatal Doppler flow velocimetry, resulted in a selection of a study population of very premature infants (average 30 weeks) that makes impossible to generalize the findings to all premature infants. However, we decided to exclude from the study intrauterine growth restricted infants whit antenatal abnormal velocimetry, for whom HM is recommended to start feeding, and we chose to test our hypothesis in a “relatively healthy” population of premature infants. Second, all the infants in our study received an amount of OMM. In particular, infants in the PF group received 31% of their enteral feeds as OMM in the two first weeks of life, and this could have positively influenced their feeding tolerance. Third, this study is not blinded. This might suggest that clinicians already felt that PF was just as good, and therefore they were more willing to continue to increase feeds. Nevertheless, the careful observation of objective criteria to reduce or withdrawal feeding should avoid this bias.

## Conclusions

Bearing in mind that the expressed OMM is the best feeding for premature infants, that a grater support for enhance lactation of mother who deliver prematurely is needed, we think that our study can contribute to the awareness that a PF can be a valid nutritional alternative for very preterm infants when OMM is unavailable or insufficient.

## Abbreviations

BW: Birthweight
FEF: Full enteral feeding
GA: Gestational age
HM: Human milk
NEC: Necrotizing enterocolitis
OMM: Own mother’s milk
PDHM: Pasteurized donor human milk
PF: Preterm formula
PMA: Postmenstrual age
PN: Parenteral nutrition
